# Evidence-based and tailored medication in type 2 diabetes: a pathway learned from clinical trials

**DOI:** 10.1186/s12933-019-0834-0

**Published:** 2019-02-28

**Authors:** Atsushi Tanaka, Koichi Node

**Affiliations:** Department of Cardiovascular Medicine, Saga University, 5-1-1 Nabeshima, Saga, 849-8501 Japan

**Keywords:** Type 2 diabetes, Cardiovascular outcome trial, Glucose-lowering agent, Dipeptidyl peptidase-4 inhibitor, Tailored medication

## Abstract

In Japan, the choice of anti-diabetic medication is officially recommended according to the patient’s glycemic condition and disease phenotype, unlike most other regions where metformin is recommended as the first-line medication. There has been an increase in the number of available glucose-lowering agents, making it necessary to select these agents based on ever-improving evidence obtained from clinical trials. For the dipeptidyl peptidase-4 inhibitor class of drugs, nine drugs are currently available on the market in Japan. Although previous cardiovascular outcome trials (CVOTs) demonstrated non-inferiority for both major adverse cardiovascular events (MACEs) and safety for some drugs of the class, the design and results of the CARMELINA trial seemed to be slightly different from earlier trials in that it showed the drugs were safe and partially effective even in patients with renal impairment. Thus, recent CVOTs on newer glucose-lowering agents have mainly focused on the major impacts of individual classes and drugs on clinical outcomes behind their glucose-lowering action. The diverse features of the classes and individual drugs may have also highlighted not only the class-effects, but also the drug-effects of glucose-lowering agents. This will lead to clinical-based evidence and assist with optimum selection of the class and/or drug for tailored medication in patients with type 2 diabetes.

An important clinical question is whether it is possible or a clinical requisite to use glucose-lowering agents within a class of drug differently in different patients. Prior to the CARMELINA study, all dipeptidyl peptidase-4 inhibitors (DPP-4is) appeared to have similar clinical impacts on non-glycemic parameters. That study demonstrated that linagliptin treatment did not increase the risk of composite major adverse cardiovascular events (MACEs) and also met the authorities’ requirements for cardiovascular safety of glucose-lowering agents [1] similar to that reported for other DPP-4is [2]. An important finding was that linagliptin was proven to be effective even in patients with long-standing type 2 diabetes (T2D) associated with renal impairment. This efficacy had not been observed in previous major studies on DPP-4is. Of further note was that the CARMELINA study also showed slight but intriguing differences in some cardiovascular and renal outcomes from those reported by previous trials.

It is noteworthy that linagliptin treatment prevented the progression of urinary albumin excretion significantly. As stated earlier, the CARMELINA trial specifically included adults with a lower estimated glomerular filtration rate (eGFR) compared to those in previous studies, with 43% of participants in the trial having at least a moderate to severe (< 45 mL/min/1.73 m^2^) decline in baseline eGFR. The mechanism of how linagliptin ameliorated the deterioration in renal function remains unclear, although it is possible its extrarenal excretion and unknown drug-specific effects may potentially have been involved in its beneficial effect on renal function [3]. However, detailed data from a head-to-head cardiovascular outcome trial (CVOT), the CAROLINA [4], comparing linagliptin with glimepiride, are still waiting. Although some pre-clinical and clinical studies demonstrated that linagliptin has unique and multiple cardiovascular protective actions [5], effects of linagliptin on vascular function as a key surrogate marker of cardiovascular risk remain to be controversial in clinical settings [6–8].

Linagliptin treatment did not increase the risk of hospitalization for heart failure (HF), but for the first time ever, was shown to be associated with a slight reduction in the risk. There appears to be a small but noticeable difference in the risk of hospitalization for HF associated with each DPP-4i relative to placebo between the CARMELINA (HR 0.90, 95% CI 0.74–1.08) [1] and SAVOR-TIMI 53 (HR 1.27, 95% CI 1.07–1.51) [9]. In addition to more severe renal impairment, the participants in the CARMELINA study had a higher frequency of a history of HF and the use of insulin than those in the SAVOR-TIMI 53 study. Although the possible risk of HF associated with DPP-4is remains controversial [10, 11], the results of the CARMELINA study enhanced clinical evidence that linagliptin treatment for a high cardiovascular and renal risk population at least did not affect the risk of HF-relate outcomes [12]. Thus, the study may strengthen the evidence for a class-effect of DPP-4is on safety, and also identify potential beneficial drug-effects for linagliptin on cardiovascular and renal systems. These findings may therefore provide important and unique evidence for linagliptin when choosing a glucose-lowering agents or even a DPP-4i.

Recent CVOTs with other classes of agent such as sodium-glucose cotransporter 2 (SGLT2) inhibitors and glucagon-like peptide-1 (GLP-1) receptor agonists, have also demonstrated unique and attractive results which may have had a major influence on decision-making in daily clinical practice regarding the choice of drug. SGLT2 inhibitors are known to reduce the risk of HF-related outcomes and delay the progression of nephropathy in a wide range of patients with T2D, while expectations of benefit on MACE appear to be higher in patients receiving secondary prevention of cardiovascular diseases [13]. On the other hand, GLP-1 receptor agonists predominantly show beneficial impacts on MACE and mortality, although this finding was not necessarily inconsistent between trials with the class [14]. Thus, recent CVOTs on newer glucose-lowering agents may have paid more attention to both the class-specific and possible drug-specific effects of those agents on cardiovascular and renal systems than that given to their glucose-lowering action. This has the potential to cause a paradigm shift in diabetes and even cardio-renal care. Furthermore, clinical trials targeting residual large populations who do not meet the artificial inclusion criteria of recent CVOTs will also reflect real-world evidence and increase the chance of applying this evidence into daily clinical practice. This could potentially lead to more appropriate decisions regarding the class and drug to be administered in a broader range of the population. These clinical trials will therefore become an important *gateway* for assisting with the selection of the class and individual drug for overall improvement in the treatment of T2D (Fig. 1). Based on the results shown in the clinical trials, we will be able to select more optimal class and drug according to the individual patients’ clinical characteristics. This would lead to the desirable selection of glucose-lowering agents and ‘Tailored Medication’ in diabetes care.

**Fig. 1:**
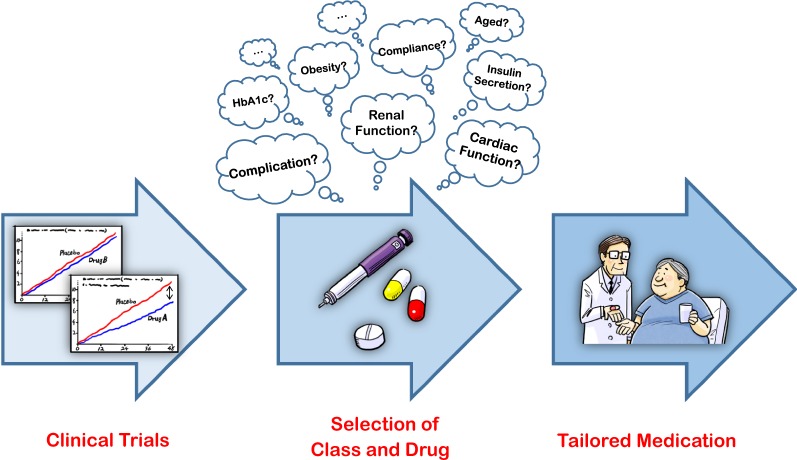
The pathway from clinical trials to selecting tailored medication for patients with type 2 diabetes

## Abbreviations

CVOT: cardiovascular outcome trial
DPP-4i: dipeptidyl peptidase-4 inhibitor
GLP-1: glucagon-like peptide-1
HF: heart failure
MACE: major adverse cardiovascular event
SGLT2: sodium-glucose cotransporter 2
T2D: type 2 diabetes
